# Impact of Nd^3+^ Substitutions on the Structure and Magnetic Properties of Nanostructured SrFe_12_O_19_ Hexaferrite

**DOI:** 10.3390/nano12193452

**Published:** 2022-10-02

**Authors:** Ashraf M. Semaida, Moustafa A. Darwish, Mohamed M. Salem, Di Zhou, Tatiana I. Zubar, Sergei V. Trukhanov, Alex V. Trukhanov, Vladimir P. Menushenkov, Alexander G. Savchenko

**Affiliations:** 1 Physical Materials Science Department, National University of Science and Technology MISiS, 119049 Moscow, Russia; 2 Physics Department, Faculty of Science, Damanhour University, Damanhour 22516, Egypt; 3 Physics Department, Faculty of Science, Tanta University, Al-Geish St., Tanta 31527, Egypt; 4 Electronic Materials Research Laboratory, Key Laboratory of the Ministry of Education & International Center for Dielectric Research, School of Electronic Science and Engineering, Xi’an Jiaotong University, Xi’an 710049, China; 5 Laboratory of Single Crystal Growth, South Ural State University, 76, Lenin Av., 454080 Chelyabinsk, Russia; 6 Laboratory of Magnetic Films Physics, SSPA “Scientific and Practical Materials Research Centre of NAS of Belarus”, 19, P. Brovki Str., 220072 Minsk, Belarus; 7 Smart Sensor Systems Laboratory, Department of Electronic Materials Technology, National University of Science and Technology MISiS, 119049 Moscow, Russia; 8 L.N. Gumilyov Eurasian National University, Nur-Sultan 010000, Kazakhstan

**Keywords:** ball milling, Halder–Wagner method, Williamson–Hall method, Nd^3+^ doping, nanohexaferrite

## Abstract

In this study, SrFe_12-x_Nd_x_O_19_, where *x* = 0, 0.1, 0.2, 0.3, 0.4, and 0.5, was prepared using high-energy ball milling. The prepared samples were characterized by X-ray diffraction (XRD). Using the XRD results, a comparative analysis of crystallite sizes of the prepared powders was carried out by different methods (models) such as the Scherrer, Williamson–Hall (W–H), Halder–Wagner (H–W), and size-strain plot (SSP) method. All the studied methods prove that the average nanocrystallite size of the prepared samples increases by increasing the Nd concentration. The H–W and SSP methods are more accurate than the Scherer or W–H methods, suggesting that these methods are more suitable for analyzing the XRD spectra obtained in this study. The specific saturation magnetization (*σ_s_*), the effective anisotropy constant (*K_eff_*), the field of magnetocrystalline anisotropy (*H_a_*), and the field of shape anisotropy (*H_d_*) for SrFe_12-x_Nd_x_O_19_ (0 ≤ *x* ≤ 0.5) powders were calculated. The coercivity (*H_c_*) increases (about 9% at *x* = 0.4) with an increasing degree of substitution of Fe^3+^ by Nd^3+,^ which is one of the main parameters for manufacturing permanent magnets.

## 1. Introduction

Historically, hexaferrite is one of the oldest materials used to make permanent magnets [1]. Since ferrites are oxide materials, hexaferrite magnets are quite resistant to corrosion and oxidation. In addition, ferrites are electrical insulators at room temperature, making them an interesting material for applications that create a lot of eddy currents inside a magnet. The raw materials are plentiful at a low price, which makes ferrites the most used material for permanent magnets [2]. Ferrites are used for applications that require high volume or weight, such as inexpensive electric motors, loudspeakers, etc., so the price should be as low as possible [3,4,5].

The doping of M-type hexaferrite (BaFe_12_O_19_, SrFe_12_O_19_) is often intended to influence the inherent magnetic structure of this compound, whose magnetic characteristics are dictated by the occupancy of five nonequivalent positions in the lattice and their magnetic coupling via oxygen-driven superexchange interactions [6]. For instance, the magnetocrystalline anisotropy can be augmented by a local increase in magnetic anisotropy due to the contribution of orbital angular momentum or changes in the crystal and chemical structures [6].

The simultaneous substitution of divalent metals with rare earth (RE) ions have been utilized to change the magnetic characteristics of hexaferrite and successfully enhance crystal anisotropy without diminishing saturation magnetization [7,8,9]. The impact of the divalent metal on the magnetic characteristics and its function in the suppression of grain increase, which can be regulated by the addition of RE metals [10,11,12], causing these alterations.

Many studies have shown that the magnetic properties of hexagonal Sr-based ferrites depend significantly on the composition and synthesis method. For example, Luo et al. [13] have reported that nanosized Nd-doped strontium ferrites, SrNd_x_Fe_12-x_O_19_, with *x* = 0.0–0.5, were successfully prepared through a chemical co-precipitation process. The crystallite size was in the range of 28–39 nm. The specific saturation magnetization (*σ_s_*) and the coercivity (*H_c_*) of strontium hexaferrite could be improved by substituting Nd^3+^ ions on Fe^3+^ ion basis sites.

The Nd^3+^-doped ferrite nanoparticles with hexagonal crystal structure were synthesized using the citrate precursor. The crystallite size ranged from 31 nm to 36 nm. The Nd^3+^ ions show a significant increase in *H_c_* and a decrease in *σ_s_* [14].

The Nd-Zn co-substituted strontium hexaferrite with the chemical formula Sr(Nd, Zn)_x_Fe_12-x_O_19_ (0.0 ≤ x  ≤  0.1) was synthesized by the sol-gel auto composition process. The XRD patterns showed the Sr hexaferrite crystal structure with crystallite size in the range from 40 nm to 50 nm. The value of *H_c_* was enhanced by the Nd and Zn substitutions. The obtained values of the residual magnetization-to-saturation magnetization ratio (*σ_r_*/*σ_s_*) indicated that prepared nanosized hexaferrite had uniaxial anisotropy [7].

M. Stingaciu et al. [15] milled commercial strontium hexaferrite powder for up to 42 hours, reducing the particle size to 400 nm. The very short time sintering process (~2 min) of spark plasma sintering at 950 °C resulted in the formation of an additional crystalline phase (Fe_3_O_4_). The resulting material revealed a change in its magnetic behavior, with an increase in maximum magnetization at 1000 Oe but a decrease in coercivity and remanence. 

J.H. Luo [16] prepared strontium hexaferrite by mechanical synthesis of a mixture of SrCO_3_ and Fe_2_O_3_, followed by annealing at 900 °C for 2 h. The saturation magnetization reached 58 emu/g, while the coercivity was 3500 Oe at room temperature.

L. Peng et al. [17] synthesized Sr_1-x_La_x_Fe_12-x_Co_x_O19 (x = 0–0.5). Ferrites were obtained by conventional ceramic techniques at 890 °C using SrCO_3_, Co_2_O_3_, La_2_O_3_, and Fe_2_O_3_ as starting materials. Hexaferrites were found to provide improved magnetic properties (*σ_s_* > 62 emu/g and *H_c_* > 4022 Oe) at x = 0.2 and 0.3.

The Co-Nd substituted M-type Sr hexaferrite with the composition Sr_1-x_Co_x_Nd_x_Fe_12-x_O_19_ (x = 0, 0.08, 0.16, and 0.24) was successfully synthesized by the ball-milling-assisted ceramic process. Sr_0.84_Co_0.16_Nd_0.16_Fe_11.84_O_19_, calcined at 1050 °C, has the highest *σ_s_* = 74.75 emu/g, and remanence (45.15 emu/g), SrFe_12_O_19_, calcined at 1150 °C, has the highest *H_c_* value (4037.01 Oe) [18]. 

However, in all cases, to obtain high hysteresis properties, the main issue is to optimize the phase structure of the SrFe_12_O_19_ (SFO) powder in the final synthesis stage. The point is that it is necessary to simultaneously solve two problems: to ensure the completion of the solid-state reaction of the formation of the SFO phase, which requires high annealing temperatures, and to prevent the formation of large multi-domain SFO grains with low coercivity. From the point of view of approaching the solution to this problem, the method of mechanochemical synthesis seems to be the most promising, besides other advantages such as its simplicity, high productivity, low cost, and well-controllable grain size compared with other methods [19]. 

Despite the large number of works devoted to studying nanocrystalline SFO powders obtained by mechanochemical synthesis and according to our knowledge, no reports available that examined the relationship between the structure and magnetic properties (theoretically and experimentally) of Nd-substituted Sr-hexaferrite (M-type) were synthesized by the ball milling followed by the calcination in the air using SrCO_3_, Nd_2_O_3_, and Fe_2_O_3_ as raw materials.

In the present study, Nd-substituted Sr-hexaferrite was obtained by mechanochemical synthesis. The microstructure, morphology, and magnetic properties of Nd-substituted Sr-hexaferrite have been studied. Using the experimental X-ray diffraction spectra data, as well as various methods for their analysis, determining the size of crystallites of the prepared powders were carried out, and the specific saturation magnetization, the effective anisotropy constant, the field of magnetocrystalline anisotropy, and the field of shape anisotropy for SrFe_12-x_Nd_x_O_19_ (0 ≤ x ≤ 0.5) powders were calculated.

## 2. Materials and Methods

### 2.1. Synthesis Procedure and Method

For the synthesis of Sr-hexaferrite powders with the nominal composition SrFe_12-x_Nd_x_O_19_, where *x* = 0, 0.1, 0.2, 0.3, 0.4, and 0.5, stoichiometric mixtures of starting materials SrCO_3_ (Reachem, purity of 99%, GOPRO Inc., San Mateo, CA, USA), Nd_2_O_3_ (Novosibirsk rare earth metals plant, 99.5%, GOPRO Inc., San Mateo, CA, USA), Fe_2_O_3_ (Vekton, 99.9%, GOPRO Inc., San Mateo, CA, USA), and 5 mL of acetone (Reachem, 99%, GOPRO Inc., San Mateo, CA, USA) were loaded into 80 mL milling vials. High-energy milling was carried out on an Activator 2S planetary ball mill (CJSC NOVIC, Novosibirsk, Russia) at a rotation speed of the disc, and the vials were 400 rpm. The ratio between the powder’s weight and the balls’ weight was 1:10. The process was carried out in the air for 6 h at room temperature. The powders obtained after high-energy milling were subjected to heat treatment (annealing) at a temperature of 1000 °C for 2 h (with a heating rate of 10 °C/min), which was carried out in a tubular resistance furnace. After annealing, the powders were cooled in the air [20].

### 2.2. Characterization Methods

An X-ray diffractometer of the DRON-4 (CJSC NOVIC, Novosibirsk, Russia), with Co-K_α_ radiation (λ = 1.7902 Å), was used for X-ray diffraction (XRD) characterization. The phase analysis was performed using the PDF-2 powder diffraction database. In addition to phase analysis, Rietveld analysis was also performed using Rigaku PDXL software (version 2.0.2.0, Rigaku Corporation, Tokyo, Japan). Corrections for Instrumental line broadening were handled by an instrumental resolution file (.irf) created based on data collection of a standard Ge-monocrystalline sample under identical conditions. The file was implemented in the profile analysis program Rigaku PDXL software. The corresponding correct line profile was extracted from a fit of the measured intensity data for the sample and standard [21]. 

The microstructure analysis by scanning electron microscopy (SEM) was performed using a Bruker AX-S Quantax 200 Scanning Electron Microscope (Bruker AXS Microanalysis GmbH, Berlin, Germany) system and transmission electron microscopy (TEM) analysis using A JEM-1400 microscope (JEOL Ltd., Tokyo, Japan). A vibrating-sample magnetometer (VSM 250, Xiamen Dexing Magnet Tech. Co., Ltd., Xiamen, China) was used to perform magnetic characterization of the synthesized powders, with a magnetizing field of 18 kOe at room temperature [22].

## 3. Results and Discussion

### 3.1. X-ray Structural Analysis and Phase Composition of SrFe_12-x_Nd_x_O_19_ (0 ≤ x ≤ 0.5) Powders

**Figure 1:**
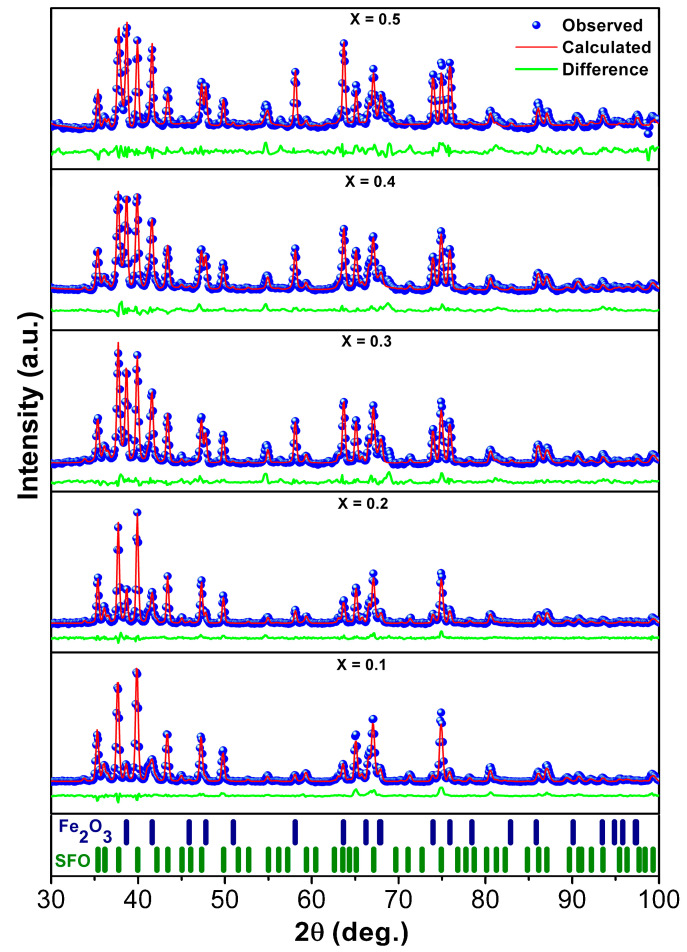
X-ray diffraction spectra of SrFe_12-x_Nd_x_O_19_ powders, where 0 ≤ *x* ≤ 0.5, and bar graphs of hexaferrite SFO and α-Fe_2_O_3_.

As can be seen in Figure 1, the phase with the hexaferrite structure SrFe_12_O_19_ (JCPDS # 80-1198) is dominant in the synthesized powders of SrFe_12-x_Nd_x_O_19_, where 0 ≤ *x* ≤ 0.5. The diffraction peaks at angle 2θ = 35.4°, 37.7°, 39.9°, 43.4°, 47.3°, and 49.8° correspond to the main diffraction planes (110), (107), (114), (203), (205), and (206) of hexagonal SFO. However, in all cases, cubic α-Fe_2_O_3_ (JCPDS # 89-0599) is present as a second phase. The diffraction planes (104), (110), (024), and (116) at 2θ = 38.65°, 41.48°, 58.06°, and 63.61° identify the α-Fe_2_O_3_ phase. The presence of α-Fe_2_O_3_ in powders of SrFe_12-x_Nd_x_O_19_ hexaferrite with Nd (*x*) content from 0 to 0.5 may be due to the incomplete reactions of Sr^2+^ and Fe^3+^ to form SFO under synthesis conditions [23].

**Figure 2:**
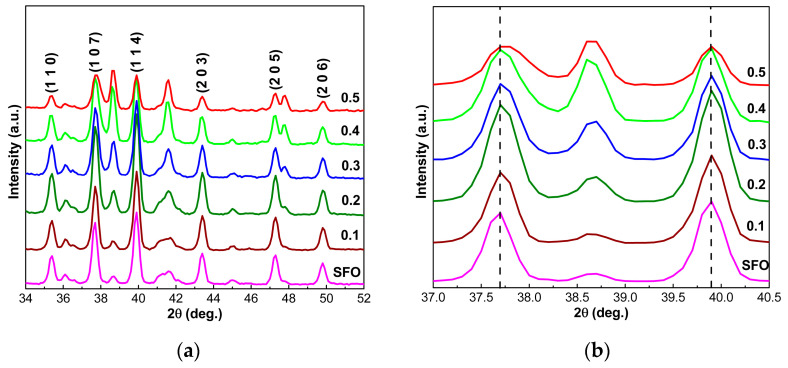
XRD spectra of hexaferrite powders SrFe_12-x_Nd_x_O_19_ (0 ≤ *x* ≤ 0.5) (**a**) and diffraction spectra fragments in the range of 2*θ* from 37° to 40.5° (**b**).

As shown in Figure 2a, the intensity of SFO-peaks (107) and (114) decreases as the concentration of Nd^3+^ ions increases. In contrast, the intensity of peaks α-Fe_2_O_3_ increases as the concentration of Nd^3+^ increases, and the α-Fe_2_O_3_ phase content increases at the expense of the M-type phase [24]. At a high concentration of dopant impurity Nd^3+^, considering the low solubility of RE ions in hexaferrite SFO, the excess introduction of Nd^3+^ leads to the formation of compound SrFeO_2.83_.

The volume fraction of phases and the Rietveld parameters (*R—*unweighted pattern (*R*_p_), *R—*weighted pattern (*R*_wp_), and goodness of fit (*χ*^2^)) of the studied SrFe_12-x_Nd_x_O_19_ (0 ≤ *x* ≤ 0.5) hexaferrite powders are given in Table 1.

### 3.2. Comparative Analysis of Crystallite Sizes of the Prepared Powders SrFe_12-x_Nd_x_O_19_ (0 ≤ x ≤ 0.5)

Using experimentally obtained XRD spectra, Figure 1, a comparative analysis of crystallite sizes in the prepared powders was carried out by different methods.

#### 3.2.1. Scherrer Method

Scherrer derived an equation for the ideal condition of a completely parallel, infinitely thin, monochromatic X-ray beam diffracting on a monodisperse, crystallite-shaped powder [25]. The crystal size and internal strain factors contribute to the widening of the diffracted Bragg peak in nanocrystals. Typically, this widening comprises one physical and one instrumental component, the latter of which can be adjusted by the following relation [26]:

(1) $$\beta_{hkl}={\left[\beta_{meas.}^{2}-\beta_{inst.}^{2}\right]}^{1/2}$$

where *β_hkl_* is the corrected peak broadening, *β_meas_*. is the measured broadening, and *β*_inst_. is the instrumental broadening. The instrumental and physical broadening of the peaks were measured in terms of the full width at half maximum (FWHM). Thus, the Scherrer method can calculate the crystallite size without considering the strain contribution. Crystallite size (*D*) and internal strain (*ε*) were calculated using the following equations:

(2) $$D=\frac{K\lambda }{\beta_{hkl}cos\theta }$$

(3) $$\varepsilon =\frac{\beta_{hkl}}{4\;tan\theta }$$

where *K* is the shape factor or morphological parameter and equal to 0.94, the wavelength (*λ*) of the X-ray is 1.791 Å for Co-K_α_ radiation, and *θ* is the peak position *θ* and *β_hkl_*, expressed in radians.

Monshi [27] proposed some modifications to the Scherrer equation. It was noticed that the Scherrer equation gives more and more overestimated values of crystallite size as the values of *d*_hkl_ (distance between the (*hkl*) diffracting planes) decrease and the values of 2*θ* increase because the product *β*cos*θ* cannot be maintained. A modification of the Scherrer equation is to determine the crystallite size for each mean peak (Equations (4) and (5)). In doing so, the error in estimating the size of crystallites is reduced, mainly.

(4) $$\beta_{hkl}=\frac{K\lambda }{Dcos\theta }=\frac{K\lambda }{D}\frac{1}{cos\theta }$$

(5) $$ln\beta_{hkl}=ln\left(\frac{K\lambda }{D}\right)+ln\left(\frac{1}{cos\theta }\right)$$

**Figure 3:**
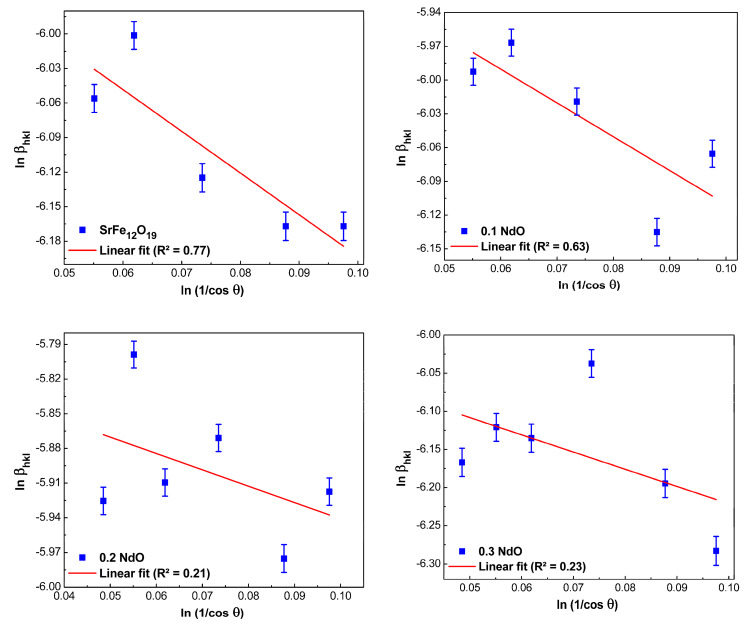

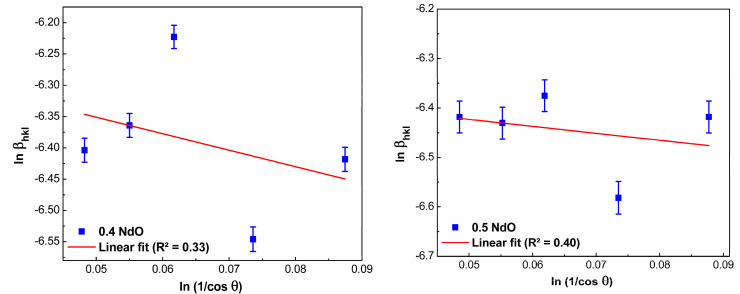
Scherrer plots for hexaferrite powders SrFe_12-x_Nd_x_O_19_ (0 ≤ *x* ≤ 0.5).

As it follows from Equation (5), in the coordinates $ln\left(\frac{1}{cos\theta }\right)$ vs. $ln\beta_{hkl}$ it should be obtained as a straight line with the slope about unity and cross the ordinate axis at the point $ln\left(\frac{K\lambda }{D}\right)$, by which the size of crystallites was calculated; see Figure 3.

As can be seen in Figure 2a, several intense lines (peaks) are observed in the range 2*θ* from 34° to 52°. All of these peaks are expected to provide identical crystallite size values. However, as seen in Figure 3, different values were obtained for each peak. At the same time, each of them had a different systematic error. After appropriate corrections, we found that as the Nd-content in SrFe_12-x_Nd_x_O_19_ powders increases from *x* = 0 to *x* = 0.5, the size of crystallites increases from 60.9 to 97.5 nm.

#### 3.2.2. The Williamson–Hall (W–H) Method

In contrast to the Scherrer formula, the W–H approach considers the impact of strain-induced widening of the diffraction lines and may be used to compute the intrinsic strain independently from the crystallite size. As stated previously, the physical diffraction line broadens due to nanocrystal size and micro deformation. Therefore, the overall expansion may be expressed as [26]:

(6) $$\beta_{total}=\beta_{size}+\beta_{strain}$$

where *β_size_* represents the expansion due to crystallite size and *β_strain_* represents the expansion due to lattice strain. The intrinsic strain influences the physical broadening of the XRD profile, which is connected to the effective stress and Bragg angle through Equation (7):

(7) $$\beta_{hkl}=\frac{K\lambda }{Dcos\theta }+4\varepsilon tan\theta$$

Equation (7) can be mathematically represented as follows:

(8) $$\beta_{hkl}cos\theta =\frac{K\lambda }{D}+4\varepsilon sin\theta$$

**Figure 4:**
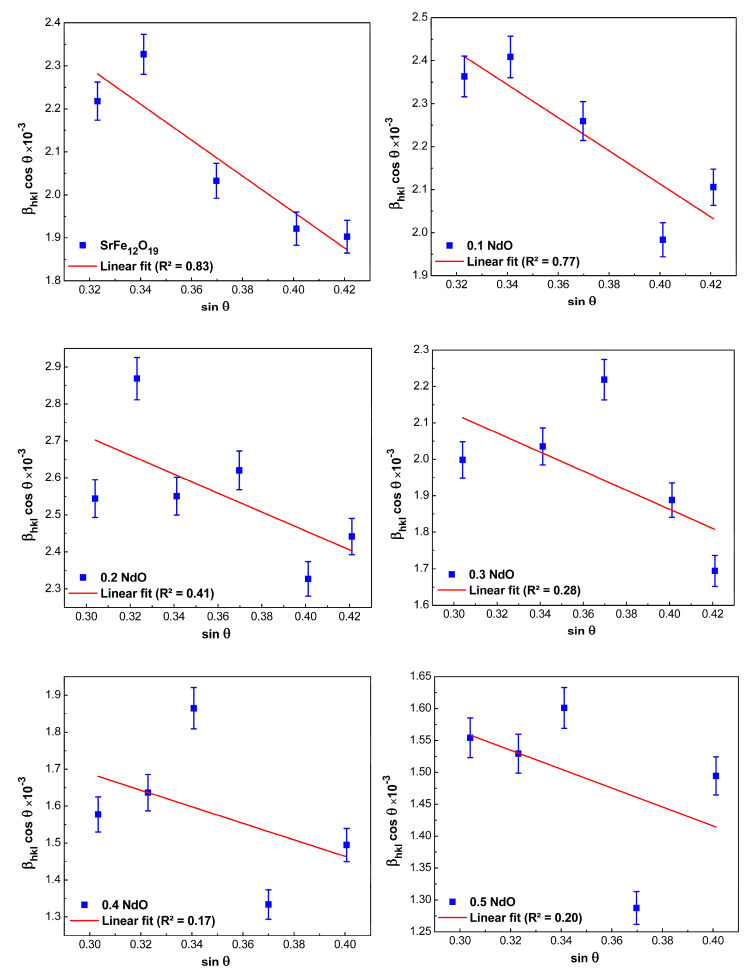
Williamson–Hall plots for hexaferrite powders SrFe_12-x_Nd_x_O_19_ (0 ≤ *x* ≤ 0.5).

According to the slope of the straight line in coordinates sinθ and $\beta_{hkl}cos\theta$, the lattice strain and the size of crystallites can be estimated by extrapolating the equation to the intersection with the Y-axis using Equation (9); see Figure 4:

(9) $$D=\frac{K\lambda }{intercept\;\left(y\right)}$$

As a result of the performed constructions on the definition of values at the crossing point and calculations, it was found that as the Nd-content increases in SrFe_12-x_Nd_x_O_19_ powders from *x* = 0 to *x* = 0.5, the size of crystallites increased from 49.4 to 89.5 nm. Due to the issue of isotropy, however, this strategy is not at all practical.

#### 3.2.3. Size-Strain Plot (SSP) Method

In several models, for example, in the size-strain plot (SSP) method, the X-ray diffraction line profiles are analyzed by representing them as a superposition of two functions: Lorentz and Gauss. In the SSP model, the dimensional broadening of the line profile is treated as a Lorentz distribution function, and the strain-induced broadening is treated as a Gaussian distribution function. The resulting line widening in this graphical method can be represented as [28]:

(10) $$\beta_{hkl}=\beta_{L}+\beta_{G}$$

where *β*_L_ is the dimensional broadening described by the Lorentz function and *β*_G_ is the broadening due to strain described by the Gauss function. The SSP method allows good results for isotropic samples at small diffraction angles (θ). However, at high values (θ), the approximation accuracy becomes unsatisfactory [29]. First of all, it is connected with a significant error in the X-ray structural analysis data at high angles caused by essential overlapping of diffraction lines.

In the SSP method, the analysis of experimental data is performed using the following equation [30]:

(11) $${\left(d_{hkl}\beta_{hkl}cos\theta \right)}^{2}=\left(K\lambda /D\right)\left(d_{hkl}^{2}\beta_{hkl}cos\theta \right)+{\left(\varepsilon /2\right)}^{2}$$

**Figure 5:**
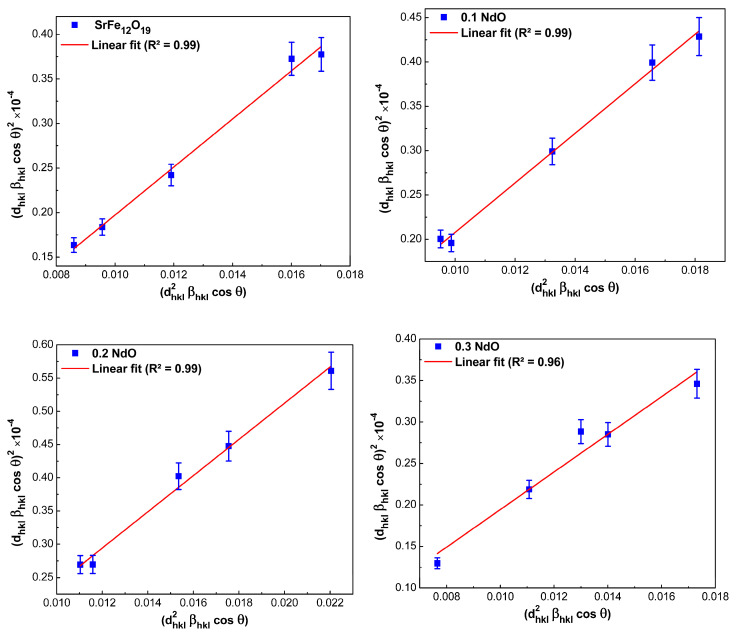

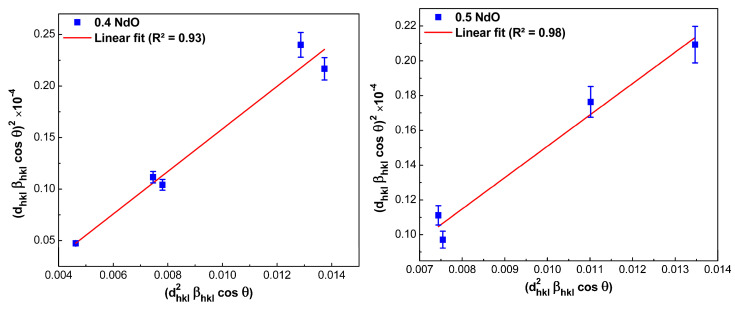
Plots in size-strain coordinates for hexaferrite powders SrFe_12-x_Nd_x_O_19_ (0 ≤ *x* ≤ 0.5).

From Equation (11), it follows that in the coordinates, ($d_{hkl}^{2}\beta_{hkl}cos\theta$) as a function of ${\left(d_{hkl}\beta_{hkl}cos\theta \right)}^{2}$ should obtain a straight line, the slope of which allows for the determining of the size of crystallites and the point of intersection with the ordinate axis to calculate the strain value of the prepared powders. The obtained results from the SSP method are shown in Figure 5. It was found that, as in the cases considered above, with increasing Nd-content in SrFe_12-x_Nd_x_O_19_ powders from *x* = 0 to *x* = 0.5, the average size of crystallites increased from 66.5 to 99.4 nm.

#### 3.2.4. Halder–Wagner (H–W) Method

In Scherer and Williamson–Hall’s methods, the broadening of X-ray diffraction lines due to the crystallite size was supposed to be the Lorentz function and the broadening due to lattice strain as the Gauss function. However, X-ray diffraction lines are not described by either the Lorentz function or the Gaussian function since the Gaussian function represents the maximum line region well. Still, its tails decay too quickly and, on the other hand, are well described by the Lorentz function, which does not fill the entire area of the Bragg diffracted peak [26].

The Halder–Wagner method assumes that the peak broadening is a symmetric Voigt function, a convolution of the Lorentz and Gauss functions. Hence, for the Voigt function, the full width at half-maximum of the physical profile in the H–W method can be written as follows:

(12) $$\beta_{hkl}^{2}=\beta_{L}\beta_{G}+\beta_{G}^{2}$$

**Figure 6:**
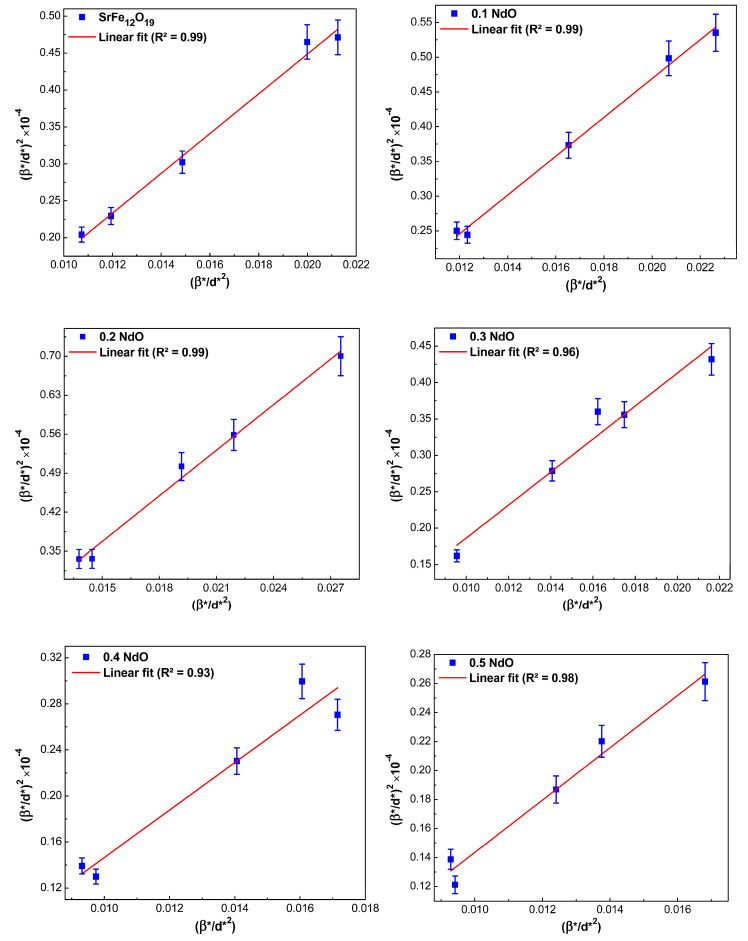
Halder–Wagner plots for hexaferrite powders SrFe_12-x_Nd_x_O_19_ (0 ≤ *x* ≤ 0.5).

This approach lends greater weight to the Bragg peaks at small and medium angles when minimal diffraction peak overlap. The formula (Equation (13)) defines the relationship between crystallite size and lattice strain according to the H–W technique [26]:

(13) $${\left(\frac{\beta^{*}}{d^{*}}\right)}^{2}=\frac{1}{D}\left(\frac{\beta^{*}}{d^{*2}}\right)+{\left(\frac{\varepsilon }{2}\right)}^{2}$$

where $\beta^{*}=\frac{\beta_{hkl}cos\theta }{\lambda }$ and $d^{*}=\frac{2sin\theta }{\lambda }$. Obviously, in the coordinates $\frac{\beta^{*}}{d^{*2}}$ vs. ${\left(\frac{\beta^{*}}{d^{*}}\right)}^{2}$, their slope should correspond to the average size of crystallites. The intersection point with the ordinate axis determines the value of the internal strain of nanocrystals, as shown in Figure 6. The average size of crystallites was also calculated.

It turned out that as the Nd-content in SrFe_12-x_Nd_x_O_19_ powders increased from *x* = 0 to *x* = 0.5, the average size of crystallites increased from 66.5 to 99.5 nm. At the same time, it can be argued that, compared with the previously considered methods, the Halder–Wagner method is more accurate (i.e., the description of broadening of diffraction lines by symmetric Voigt functions seems more realistic); Figure 6 clearly shows a good agreement between the approximating straight lines and the experimentally obtained points.

#### 3.2.5. Comparison of the Average Crystallite Sizes Obtained by Different Methods

The average crystallite size values that were calculated by different methods for all the prepared samples (SrFe_12-x_Nd_x_O_19_, where 0 ≤ *x* ≤ 0.5) are given in Table 2. From Table 2, we can conclude that all the methods used to analyze the X-ray diffraction spectra agree that the average crystallite sizes increase by increasing the Nd-concentration. As for the question of which of the methods is preferable, i.e., it allows describing experimentally obtained spectra more accurately to answer it, let us turn to the obtained values of correlation coefficient (*R*²)—they can serve as one of the indirect parameters for differentiation of all studied linear methods. In this case, the method can be considered more accurate if *R*² differs from 1 or, in other words, if the experimentally obtained points are located directly or very close to the approximation line [31]. By this criterion and as shown in Figure 3 to Figure 6, the H–W and SSP methods are more accurate than the Scherer or W–H methods, suggesting that these methods are more suitable for the analysis of the XRD spectra obtained in our study.

**Figure 7:**
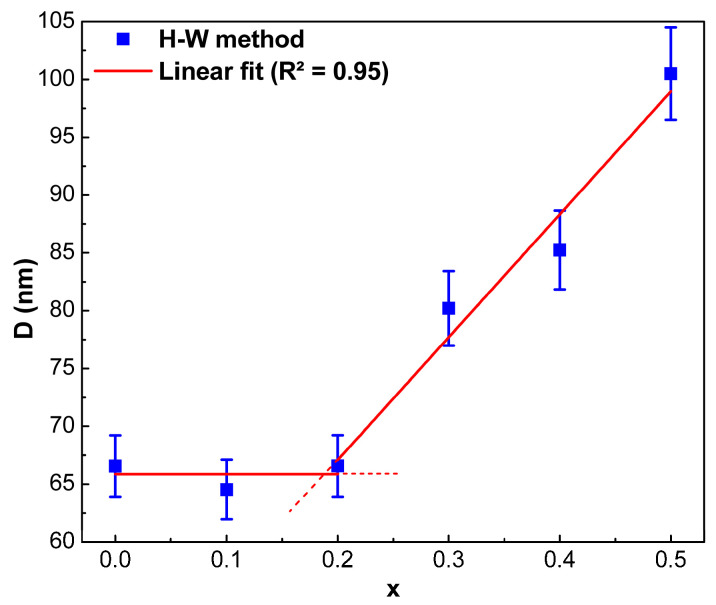
Results of approximation of sizes of crystallites in SrFe_12-x_Nd_x_O_19_ powders, where 0 ≤ *x* ≤ 0.5, obtained by the Halder–Wagner method as a function of Nd content (x).

The crystallite size of SrFe_12-x_Nd_x_O_19_ hexagonal ferrite powders was analyzed as a function of Nd content (*x*), as shown in Figure 7, which was plotted using the values obtained by the Halder–Wagner method. As shown in Figure 7, two distinct linear trends in *D* were noticed, strongly dependent on the composite’s Nd content (x). From *x* = 0 to 0.2, *D* does not change significantly with increasing Nd^3+^ content until *x* = 0.2, which is given in the linear fit equation, *D* = 0.01*x* + 65.88. This may be related to the lattice strain created by Nd^+3^, up to *x* = 0.2, which can be absorbed (dissipated) in the crystal structure of hexaferrite grains without changing its type. In the range 0.2 < *x* ≤ 0.5, *D* increases with increasing Nd^3+^ content according to the linear fit equation: *D* = 102.7*x* + 47.09. This may be because the lattice cannot “absorb” all rising strain, and, as a consequence, strain grows in it, the lattice of hexaferrite becomes unstable, and large crystallites of SrFeO_2.83_ phase appear, and this new phase percentage increases with increasing *x* (Table 1). As it is known, limited solubility in the matrix phase, the substituents/alloying elements are always located at or near the grain surface to minimize the elastic energy. Suppose the grain size increases; however, the surface to volume ratio decreases, which reduces the available grain surface area for grain “adsorption”. As a result, the system’s energy is reduced by forming a new phase, compensating for the excess elastic energy that increases as the concentration of the doping element increases [32].

#### 3.2.6. Lattice Parameters of SrFe_12-x_Nd_x_O_19_ Powders (0 ≤ x ≤ 0.5)

XRD determined the lattice constants of the synthesized compounds [33]. At the same time, it was taken into account that near the surface of crystallites, the lattice constants are affected by surface defects, which may slightly deviate their magnitude from the standard values [34]. The lattice constants of hexaferrite powders can be calculated by knowing the wavelength of the X-ray diffractometer source and the interplanar distance [34]. This distance is determined by the law of diffraction (Bragg’s law):

(14) $$n\lambda =2d_{hkl}\;sin\theta$$

where *n* is the number of reflection orders. The lattice constants (*a, c*) of the hexaferrite powders can be obtained using the following formula [35]:

(15) $$\frac{1}{d_{hkl}^{2}}=\frac{4}{3}\left(\frac{h^{2}+hk+k^{2}}{a^{2}}\right)+\frac{l^{2}}{c^{2}}$$

The unit cell volume (*V*_*cell*_) of the phase with a hexagonal structure is calculated by the following formula [35]:

(16) $$V_{cell}=\frac{\sqrt{3}}{2}a^{2}c$$

The lattice parameters (*a*, *c*), the ratio (*c/a*), as well as the unit cell volumes of the phase with the SrFe_12_O_19_ hexaferrite structure in the synthesized powders SrFe_12-x_Nd_x_O_19_, where 0 ≤ *x* ≤ 0.5, calculated using the Formula (15) and (16) are shown in Table 3. At the same time, the most intense main lines, according to which the calculation was carried out, are shown in Figure 2b.

As shown in Figure 2b, the position of the lines does not change as the Nd-concentration in SFO hexaferrite increases up to *x* = 0.3. However, with a further increase in Nd-concentration, the maxima of the lines are shifted towards larger angles. As seen in Table 3, the lattice parameters *a* and *c* slightly fluctuate with irregular changes in their values. In particular, we assume that the Nd^3+^ substitute is mainly the Fe^3+^, but there is a nonzero probability that some Nd^3+^ substitutes for Sr^2+^ cause fluctuations in lattice parameters [36,37].

Nevertheless, from the results given in Table 3, it is clear that for powders with Nd- concentration *x* > 0.4, a slight shift of diffraction peaks towards higher angles is observed (see Figure 2b). However, we have already mentioned that at low concentrations of the doping element, its presence is limited to near-surface layers of grains. In this case, the probability of lattice phase parameter change is tiny, although the appearance of weak asymmetry of diffraction lines is possible. As a confirmation of the above, the diffraction spectra of compounds SrFe_12-x_Nd_x_O_19_ at *x* ≤ 0.3 has been observed. Such Nd-content can be considered as the upper limit of the concentration of this doping element [32]. If the degree of doping exceeds this limit, the excess atoms of the alloying element are “smeared” over the volume of the phase or make the hexaferrite phase unstable, transforming it into a new phase. As follows from the results in Table 1, the structure of powders exhibit large crystallites of the SrFeO_2.83_ phase, whose quantity increases with increasing *x*. At the same time, in the first case, due to the small average Nd-concentration volume, we do not see essential changes in the lattice parameter of the hexaferrite phase [32].

According to T.R. Wagner [38], analysis of the *c*/*a* ratio can be used for quantitative estimation of the structure type because the structure of M-type hexaferrite remains stable if the ratio is less than 3.98. From the results given in Table 3, it is seen that the values of the *c*/*a* ratio for the phase with SrFe_12_O_19_ hexaferrite structure in the prepared powders of nominal composition SrFe_12-x_Nd_x_O_19_, where 0 ≤ *x* ≤ 0.5, are in the range from 3.919 to 3.925, which corresponds to the stability region of the phase with the M-type hexaferrite structure. The latter is also confirmed by the fact that the unit cell volume of the phase with the hexaferrite structure SrFe_12_O_19_ (Table 3) in the prepared powders changes insignificantly due to its stronger dependence on the lattice parameter (*a*), which is almost not affected by the replacement of Fe^3+^ by Nd^3+^ [39].

#### 3.2.7. Surface Morphology of SrFe_12-x_Nd_x_O_19_ (0 ≤ x ≤ 0.3) Powders

**Figure 8:**
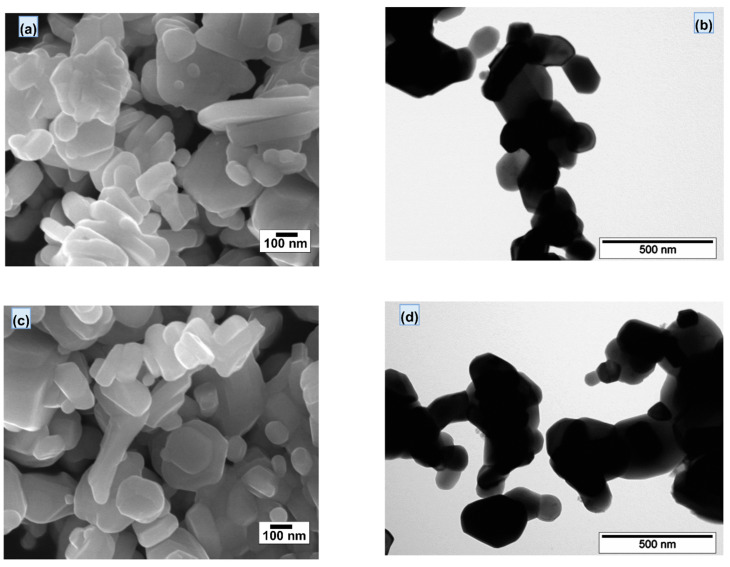
Scanning electron microscopy (SEM) (**a**,**c**) and transmission electron microscopy (TEM) (**b**,**d**) microphotographs obtained for SrFe_12-x_Nd_x_O_19_ powders with *x* = 0 (**a**,**b**) and 0.3 (**c**,**d**), respectively.

Scanning and transmission electron microscopy have studied the particle size and morphology of SrFe_12-x_Nd_x_O_19_ powders, where 0 ≤ *x* ≤ 0.5. For example, in Figure 8, the SEM and TEM images of powders with *x* = 0 and *x* = 0.3 are shown. The SEM microstructure study showed that all powders consist of hexagonal lamellar particles (consistent with their hexagonal structure), with cross-sectional dimensions from 200 to 300 nm. All samples had aggregated grains because nanocrystals tend to achieve a lower energy state by minimizing their interfaces with neighboring particles [40]. As shown in Figure 8c, as the ratio of substituent ions increases from *x* = 0 to *x* = 0.3, the particle size seems to become a little larger, which is consistent with the XRD results (Table 2). Some small spherical particles on the surface of agglomerated particles were observed in the sample, which may be the abundant secondary Fe_2_O_3_ phase, which was also shown in the XRD spectrum (Figure 1).

The difference in the calculated particle size values from XRD and SEM was attributed to the XRD peak broadening occurring from the coherent scattering of crystalline domains. At the same time, the SEM and TEM images show the total attenuation from the sample, independently of the coherency of the domains and crystallinity. Therefore, we conclude that each particle observed in SEM is formed by the aggregation of several crystallites [19,41].

### 3.3. Magnetic Properties of SrFe_12-x_Nd_x_O_19_ (0 ≤ x ≤ 0.5) Powders

**Figure 9:**
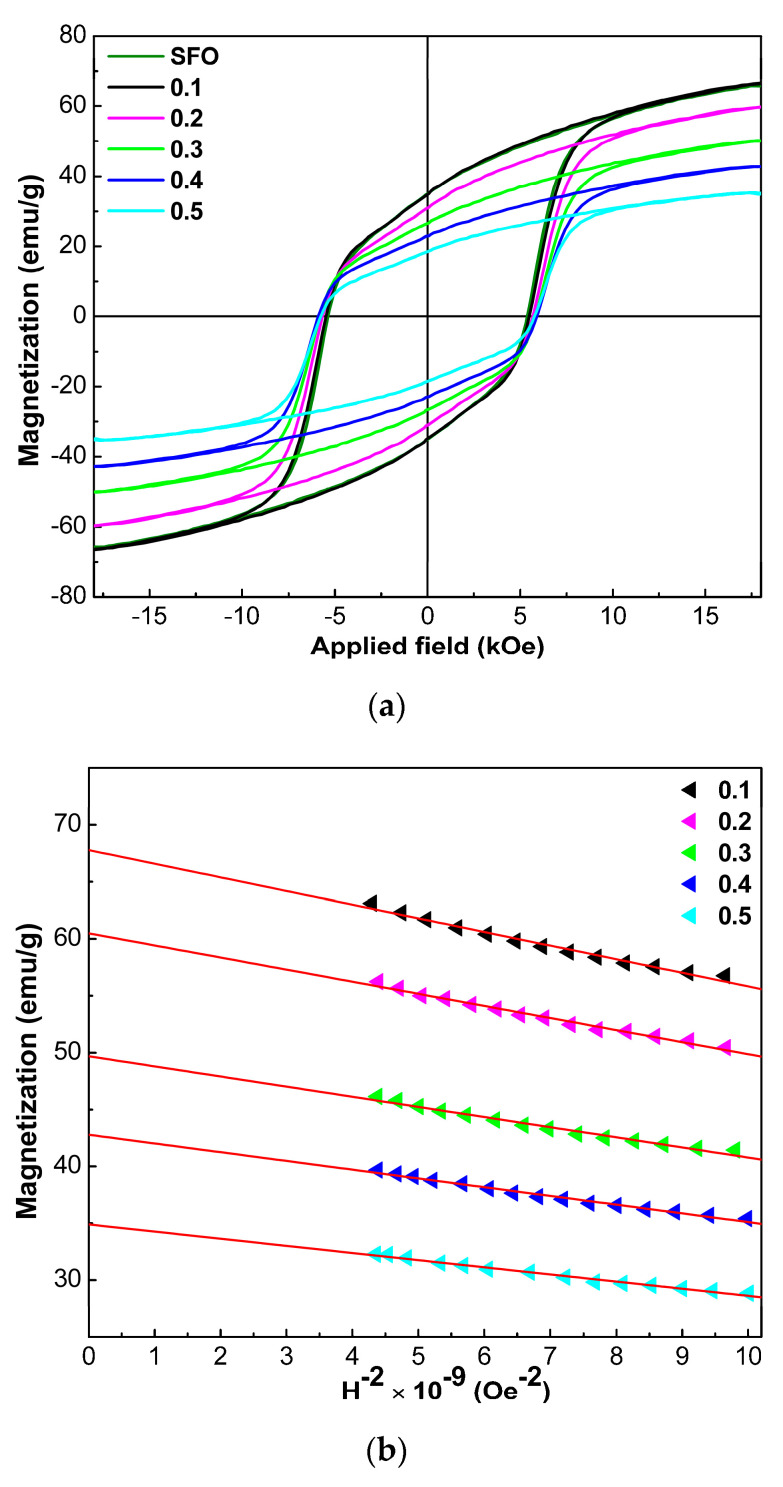
Magnetic hysteresis loops measured at room temperature (**a**) and linearized plots of the dependence of magnetization *σ* on *H*^−2^ (**b**) SrFe_12-x_Nd_x_O_19_ (0 ≤ *x* ≤ 0.5) powders.

Figure 9a shows the magnetic hysteresis loops of the prepared powders of the nominal composition SrFe_12-x_Nd_x_O_19_, where 0 ≤ *x* ≤ 0.5, measured at room temperature.

The values of coercivity (*H_c_*), the maximum corresponding magnetizations due to the applied field of 18 kOe (*σ*_18_), specific residual magnetization (*σ_r_*), and the value of the ratio *σ_r_*/*σ*_18_ are given in Table 4.

The hysteresis loops in Figure 9 show that the specific magnetization of all the studied powders does not reach saturation, even in the 18 kOe. Magnetization reversal in strong fields is performed by rotating the magnetic moments of individual particles [42]. Considering the law of approximation to saturation, W.F. Brown Jr. [43] proposed to use the following expression to estimate the values of specific saturation magnetization (*σ_s_*):

(17) $$\sigma =\sigma_{s}\left(1-\frac{b}{H^{2}}\right)$$

where *σ* is the magnetization, *H* is the applied magnetic field, and *b* is the parameter related to the magnetocrystalline anisotropy.

Figure 9b shows the dependence of magnetization values on 1/*H*^2^. Considering Equation (17), the *σ_s_* was determined from the point of intersection of the straight line with the Y-axis (Figure 9b). In this case, the slope of the corresponding lines is equal to the product of *σ_s_* and *b* [44]. Therefore, having determined *σ_s_*, we can determine the value of the parameter *b*. Knowing the parameter *b* and using the approximate equation for uniaxial magnetic nanoparticles, the value of the effective anisotropy constant (*K_eff_*) can be determined [45]:

(18) $$K_{eff}=\sigma_{s}{\left(\frac{15b}{4}\right)}^{0.5}$$

For Sr-hexaferrite, under the assumption that the hexagonal *c* axis is the easy magnetization axis, B.D. Cullity [46] found that the *K_eff_* of SrFe_12_O_19_ is slightly larger than 3.3 × 10^6^ erg/cm^3^ (or 6.22 × 10^5^ erg/g). For SrFe_12_O_19_, we obtained *K_eff_* = 5.25 × 10^5^ erg/g, which is in good agreement with the results of B.D. Cullity.

As known, Sr-hexaferrite owes its magnetic hardness to magnetocrystalline anisotropy [47]. Therefore, our *K_eff_* values (Table 5) for SrFe_12-x_Nd_x_O_19_ powders can be associated with uniaxial magnetocrystalline anisotropy in a significant part. It should be noted that, in the case of nanopowders, we are at least dealing with two types of magnetic anisotropy fields: *H_a_* and *H_d_*, where *H_a_* is the field of magnetocrystalline anisotropy, and *H_d_* is the field of shape anisotropy, which can be defined by the expression [48]:

(19) $$H_{d}=N_{d}\sigma_{s}$$

where *N_d_* is the demagnetization coefficient, and taking into account the shape of the obtained particles, the shape anisotropy field lies in the basal plane [41]. To calculate the *H_a_*, the following equation was used [46]:

(20) $$H_{a}=\frac{2K_{eff}}{\sigma_{s}}$$

The values of *H_d_* and *H_a_* obtained using Equations (19) and (20) are given in Table 5. Note that the shape and size of grains play an important role in determining the coercivity of hard magnetic materials.

According to the Stoner–Wohlfarth theory for non-interacting single-domain particles, for the coercivity of highly anisotropic hexaferrite powders, the *H_C_* can be written as follows [49]:

(21) $$H_{c}=0.48\left(H_{a}-H_{d}\right)$$

Substituting Equations (19) and (20) into Equation (21), the coercive force can be obtained [50]:

(22) $$H_{c}=0.48\left[\left(\frac{2K_{eff}}{\sigma_{s}}\right)-N_{d}\sigma_{s}\right]$$

Using the obtained experimental values of *H_c_*, listed in Table 4, as well as calculated values *σ_s_* and *K_eff_*, listed in Table 5, the values of *H_d_* can be determined, and using Equation (19)—demagnetization factors of particles in SrFe_12-x_Nd_x_O_19_ powders, where 0 ≤ *x* ≤ 0.5. As it is seen from the results obtained in Table 5, *H_d_*, which depends only on the particle shape, is in a very narrow range of values (3.90 to 4.58 kOe), whereby: (1) in absolute value, the *H_d_* values are almost four times less than the corresponding *H_a_* values, that is, the magnetocrystalline anisotropy of the Sr-hexaferrite phase makes the main contribution to the magnetic hardening of the synthesized SrFe_12-x_Nd_x_O_19_ powders; (2) the demagnetization coefficient (demagnetization factor) of SrFe_12-x_Nd_x_O_19_ nanoparticles increases from *N_d_* = 68.3 to 69.76 Oe^2^ ⋅g/erg for *x* = 0 and 0.2 to *N_d_* = 116.31 Oe^2^ ⋅g/erg for *x* = 0.5, i.e., almost two times, which may indicate an increase in the aspect ratio (the ratio of particle diameter to their thickness).

**Figure 10:**
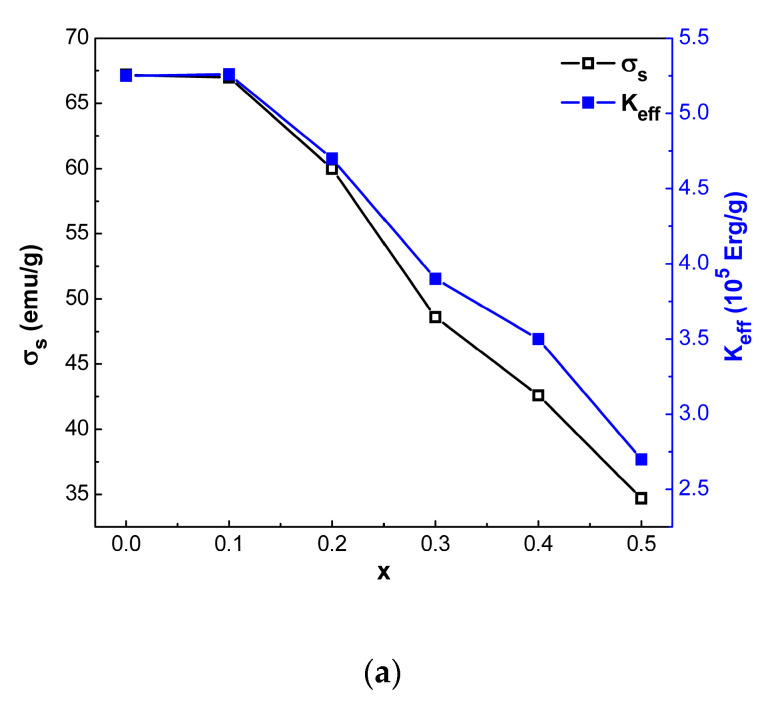

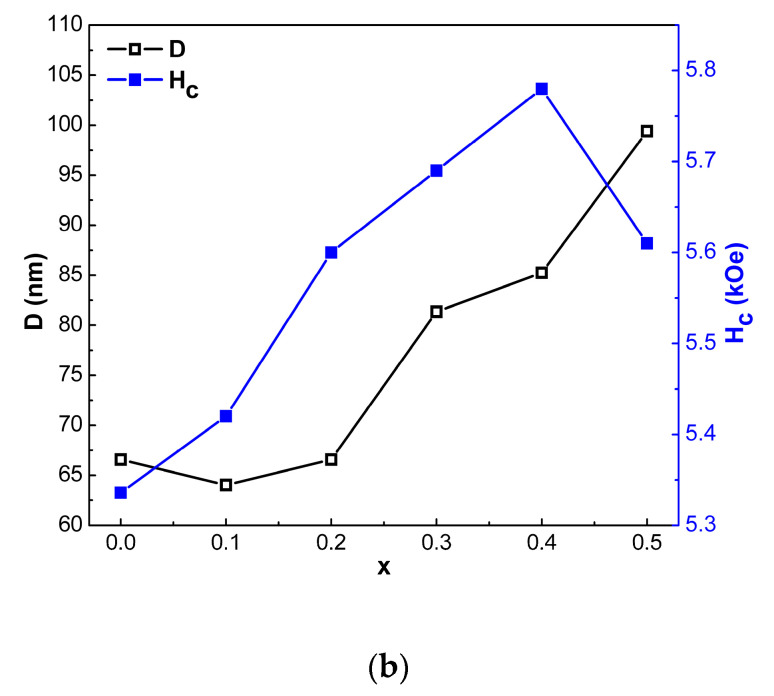
Variation of saturation magnetization and magnetic anisotropy constant (**a**) and crystallite size and coercivity as a function of Nd (*x*) content (**b**) for hexaferrite powders.

From the results given in Table 5 and Figure 10, it can be seen that the *σ_s_* of SrFe_12-x_Nd_x_O_19_ powders, where 0 ≤ *x* ≤ 0.5, decreases faster than the effective magnetic anisotropy constant (*K_eff_*) as *x* increases, which, following Equation (20), the magnetic anisotropy field strength *H_a_* and, consequently, the coercivity of powders should increase [14].

Indeed, Figure 10 shows that *σ_s_* of SrFe_12-x_Nd_x_O_19_ powders decrease, and the coercivity increases (about 9% at *x* = 0.4) with an increasing degree of substitution of Fe^3+^ by Nd^3+^. However, in the general case, the increase in *H_c_* of SrFe_12-x_Nd_x_O_19_ powders when the Nd (x) content is increased from 0 to 0.5 depends on many factors. In particular, the increase in *H_c_* can be explained by the following three reasons:

(1) According to Equation (22), the decrease of *σ_s_* with the increase of Nd (*x*) content must lead to the rise in *H_c_* [23].
(2) Correlations of *H_c_* with particle size [51]. The increase in particle size with increasing Nd (*x*) content, as seen in Figure 10(b), leads to the rise in *H_c_*, but in this case, under consideration, it is necessary to take into account the correlation in changes of *H_c_* (*x*) with the integral parameter—the field of anisotropy of the form *H_d_* (*x*).
(3) The presence of secondary phases. As can be seen from the results given in Table 1, magnetic powders with Nd (*x*) content from 0 to 0.5 contain particles of antiferromagnetic α-Fe_2_O_3_ as a second phase, which, taking into account the pinning of domain walls as the dominant mechanism of magnetization reversal in relatively weak fields, as well as an increase in the volume content of the α-Fe_2_O_3_ phase with increasing *x*, should lead to an increase in *H_c_*.

The specific saturation magnetization *σ_s_* for pure SrFe_12_O_19_ at *x* = 0 is 67.2 emu/g, which is greater than the values obtained for SrFe_12-x_Nd_x_O_19_ powders doped with Nd^3+^ and given in Table 5. It can be assumed that the following reasons can cause the decrease of *σ_s_* value in the case of doping:

(1) The presence of the impurity phase α-Fe_2_O_3_ in powders, as discussed in Section 3.1 (Table 1).
(2) The appearance of Nd^3+^ in the SrFe_12_O_19_ lattice leads to local stresses that can cause disorder in the orientation of magnetic moments, such as the appearance of local non-collinearity of magnetic moments [14].
(3) Replacing each Fe^3+^ (5 *μ_B_*) with Nd^3+^ (3 *μ_B_*) reduces the resulting magnetic moment by 2 *μ_B_* and hence can lead to a reduction in specific saturation magnetization [14].

The decrease in *σ_r_* is due, firstly, to the same reasons that cause the decrease in *σ_s_* [52], such as the presence of antiferromagnetic α-Fe_2_O_3_ and the weakly magnetic SrFeO_2_._83_ phase [53], and, secondly, the factors we also discussed above that lead to a decrease in *H_c_* at x = 0.5.

The values of the ratio *σ_r_*/*σ*_18_ for the synthesized powders SrFe_12-x_Nd_x_O_19_ are given in Table 4. It follows from Stoner–Wohlfarth theory that if this ratio is greater than 0.5, the material consists of single domain particles interacting in some way (through exchange and/or dipole interaction), and if less than 0.5, then any magnetic interaction between particles is absent, and/or powders are multi-domain [54,55]. For the investigated powders, the value of the ratio *σ_r_*/*σ*_18_ varied from 0.536 to 0.520, indicating that the hard magnetic phase powders were obtained [56,57,58]. SrFe_12-x_Nd_x_O_19_ based on substituted strontium hexaferrite is represented by single-domain crystallites, which interact with each other in exchange [59,60,61,62].

## 4. Conclusions

As a result of comprehensive studies of the effect of Nd^3+^ substitutions on the phase composition, structure, particle morphology, and magnetic hysteresis properties of SrFe_12-x_Nd_x_O_19_, where 0 ≤ x ≤ 0.5, synthesized by high-energy milling of high-purity SrCo_3_, Nd_2_O_3_, and Fe_2_O_3_, taken in the required proportion, it was found that:

(1) The Halder–Wagner (H–W) and Size-strain plot (SSP) methods are more accurate than the Scherer or Williamson–Hall (W–H) methods, suggesting that these methods are more suitable for the analysis of the X-ray diffraction spectra obtained in our study.
(2) The specific saturation magnetization *σ_s_* of SrFe_12-x_Nd_x_O_19_ powders decreases and the coercive force increases (about 9% at *x* = 0.4) with increasing degree of Fe^3+^ substitution by Nd^3+^.
(3) The values of the ratio *σ_r_*/*σ*_18_ for the synthesized powders SrFe_12-x_Nd_x_O_19_ varied from 0.536 to 0.520, indicating that the obtained powders SrFe_12-x_Nd_x_O_19_, the hard magnetic phase based on substituted strontium hexaferrite, are represented by single domain crystallites interacting with each other.
(4) For strontium hexaferrite, the effective anisotropy constant, *K_eff_* = 5.25 × 10^5^ erg/g, agrees with the results of B.D. Cullity (6.22 × 10^5^ erg/g).

## Figures and Tables

**Table 1:** The volume fraction of phases and Rietveld parameters of X-ray diffraction spectra of SrFe_12-x_Nd_x_O_19_ powders, where 0 ≤ *x* ≤ 0.5.

*x*	Phase Composition, %			Fitting Parameters		
	SrFe_12_O_19_	α-Fe_2_O_3_	SrFeO_2.83_	*R*_p_	*R*_wp_	*χ* ^2^
0	95	5	-	3.26	4.61	3.12
0.1	94	6	-	3.33	5.34	2.56
0.2	90	10	-	2.69	3.65	3.17
0.3	75	23	2	3.61	4.98	4.01
0.4	60	35	5	2.4	3.13	2.52
0.5	36	55	9	4.18	5.56	3.01

**Table 2:** The average crystallite size values for all prepared samples by different methods.

*x*	Sherrer Plot	W–H Plot	SSP	H–W Plot
0	60.92	49.41	66.51	66.51
0.1	59.71	47.72	63.93	63.29
0.2	59.12	51.14	66.31	66.31
0.3	72.21	63.93	77.82	81.36
0.4	88.56	77.82	89.52	85.24
0.5	97.48	89.51	99.41	99.41

**Table 3:** Calculated values of lattice parameters (*a*, *c*), ratios (*c*/*a*), and volumes of unit cell phases with SrFe_12_O_19_ hexaferrite structure in synthesized powders SrFe_12-x_Nd_x_O_19_, where 0 ≤ *x* ≤ 0.5.

*x*	*c* (Å)	*a* (Å)	*V* (Å^3^)	*c*/*a*
0	23.12	5.891	695	3.925
0.1	23.08	5.888	693	3.92
0.2	23.08	5.889	693.3	3.92
0.3	23.07	5.891	693.5	3.917
0.4	23.09	5.889	693.6	3.922
0.5	23.07	5.886	692	3.919

**Table 4:** Magnetic hysteresis properties of SrFe_12-x_Nd_x_O_19_ (0 ≤ *x* ≤ 0.5) powders.

*x*	*H_c_* (kOe)	*σ*_18_ (emu/g)	*σ_r_* (emu/g)	*σ_r_*/*σ*_18_
0	5.33	65.81	34.97	0.531
0.1	5.42	66.62	34.80	0.522
0.2	5.61	59.81	31.12	0.520
0.3	5.69	50.14	26.59	0.530
0.4	5.78	42.81	22.95	0.536
0.5	5.61	35.23	18.51	0.525

**Table 5:** Calculated values *σ_s_*, *K_eff_*, *H_a_*, *N_d_*, and *H_d_* for SrFe_12-x_Nd_x_O_19_ (0 ≤ *x* ≤ 0.5) powders.

*x*	*σ_s_* (emu/g)	*σ_s_⋅b* (Oe^2^ ⋅emu/g)	*K_eff_* (10^5^ erg/g)	*H_a_* (kOe)	*N_d_* (Oe^2^ ⋅g/erg)	*H_d_* (kOe)
0	67.20	1.10 × 10^9^	5.25	15.63	68.30	4.58
0.1	67.10	1.10 × 10^9^	5.25	15.65	65.69	4.39
0.2	60.01	0.99 × 10^9^	4.71	15.73	69.76	4.18
0.3	48.61	0.85 × 10^9^	3.92	16.15	81.89	4.04
0.4	42.62	0.77 × 10^9^	3.50	16.46	91.60	3.90
0.5	34.70	0.61 × 10^9^	2.82	16.27	116.31	4.03

## Data Availability

Not applicable.
